# PEO-*b*-PPO star-shaped polymers enhance the structural stability of electrostatically coupled liposome/polyelectrolyte complexes

**DOI:** 10.1371/journal.pone.0210898

**Published:** 2019-01-17

**Authors:** Camille E. Pinguet, Esther Ryll, Alexander A. Steinschulte, Jón M. Hoffmann, Monia Brugnoni, Andrey Sybachin, Dominik Wöll, Alexander Yaroslavov, Walter Richtering, Felix A. Plamper

**Affiliations:** 1 Institute of Physical Chemistry, RWTH Aachen University, Aachen, Germany; 2 Department of Chemistry, M.V. Lomonosov Moscow State University, Moscow, Russian Federation; 3 Institute of Physical Chemistry, TU Bergakademie Freiberg, Freiberg, Germany; University of Hyderabad, INDIA

## Abstract

We propose a strategy to counteract the salt-driven disassembly of multiliposomal complexes made by electrostatic co-assembly of anionic small unilamellar liposomes and cationic star-shaped polyelectrolytes (made of quaternized poly(dimethylaminoethyl methacrylate) (*q*PDMAEMA_100_)_3.1_). The combined action of (*q*PDMAEMA_100_)_3.1_ and a nonionic star-shaped polymer (PEO_12_-*b*-PPO_45_)_4_, which comprises diblock copolymer arms uniting a poly(ethylene oxide) PEO inner block and a poly(propylene oxide) PPO terminal block, leads to a stabilization of these complexes against disintegration in saline solutions. Hereby, the anchoring of the PPO terminal blocks to the lipid bilayer and the bridging between several liposomes are at the origin of the promoted structural stability. Two-focus fluorescence correlation spectroscopy verifies the formation of multiliposomal complexes with (PEO_12_-*b*-PPO_45_)_4_. The polyelectrolyte and the amphiphilic polymer work synergistically, as the joint action still assures some membrane integrity, which is not seen for the mere (PEO_12_-*b*-PPO_45_)_4_—liposome interaction alone.

## Introduction

Small unilamellar liposomes (spherical bilayer lipids vesicles) are used in many disciplines for various applications: cosmetics [1], food industry [2], pharmaceutics [3]… The function of liposomes as carriers has been extensively exploited particularly for cancer therapy [4]. Liposomes have the advantage to encapsulate hydrophobic substances in the lipidic membrane and hydrophilic substances in the cavity [5, 6]. Then, stimuli like temperature or pH variation can cause a triggered release [7, 8]. To increase their specific performance, liposomes can be modified. As an example, coatings can increase the stability of the encapsulated compound [9] and maintain its activity [10, 11]. More specifically, addition of poly(ethylene oxide) PEO chains as a liposome coating improves the circulation time [12]. However, increasing the chain length of PEO enhances the permeability of the membrane and, thus, causes leakage due to the phase separation of the liposomal bilayer membrane [13]. In contrast, PEO-dendron phospholipids owing to their increase in the liposomal stability make the liposomes stealthier [14].

Taking now facile noncovalent/nonionic attachment strategies for PEO chains, the adsorption of polymers into lipid membranes has already been observed for Pluronic F127 (PEO_98_-*b*-PPO_67_-*b*-PEO_98_) [15] and for Poloxamer P188 (PEO_80_-*b*-PPO_27_-*b*-PEO_80_) [16] for example. The polymer interactions with lipid membranes can be affected by several factors, including the degree of polymerization, architecture, hydrophobicity of the polymer, the polymer-to-lipid ratio and the incubation time [17, 18]. Simulations indicated that the PEO gets adsorbed first on the hydrophilic surface of the membrane. This facilitates the approach of the PPO block to get close to the bilayer surface. Second, while the PEO remains close to the head groups of the lipid bilayer, the PPO starts penetrating inside [19]. Hence, PEO-*b*-PPO copolymers can be used as an alternative to PEO-lipids, since the PEO block is hydrophilic and stays in water away from the head region and the PPO block goes inside the lipid membrane [20]. The binding percentage between PEO-PPO polymers and liposomes depends of the concentration in polymer and a limiting factor is the maximum surface coverage [21].

This approach can be extended to multivalent binding. Amphiphilic copolymers with several PPO moieties are expected to bind to several liposomes. While such a non-electrostatic co-assembly of ready-made liposomes is worthwhile to be investigated as such, we expect that multiliposomal carriers are built, which would allow a possible increase in the encapsulation capacity. The loading of a single liposome is limited and cannot necessarily be enhanced by simple enlargement of the single liposome. This is especially true for loading of hydrophobic substances into the lipid double layer. Combining different small liposomes would substantially increase the hydrophobic domain (see S1 File, page 4), while still keeping a reasonable overall size for successful transport through e.g. blood vessels. As a further advantage, such multiliposomal systems could contain multiple and even possible conflicting substances, which makes them considered as a magic bullet for drug delivery [22].

There are different approaches for the generation of multicompartmental constructs. E.g. the generation of micrometer-sized multicompartment liposomes can be achieved by microfluidic techniques [23]. Similarly, polymersomes with multiple compartments can be fabricated by using double emulsions with different morphology as templates [24]. To form multicompartmental liposomes, it is also possible to encapsulate small liposomes in bigger liposomes (vesosomes) or to encapsulate dendrimers in liposomes (dendrosomes) [25]. Aiming to preserve the activity of an enzyme, multicompartment carriers can be constructed of liposomes and gold nanoclusters within a polymer carrier capsule [26]. As a very facile alternative, the electrostatic coupling of several anionically charged liposomes with (single) polyions (positively charged spherical brushes [27], polyelectrolyte stars [28–30], microgels [31]…) was successfully considered, while this complexation keeps the liposomes intact [32]. Advantageously, the size of multicontainers containing several polycations can be adjusted by introducing polymer branching and PEO moieties, as shown earlier [33]. However, salt will have an effect on the multicompartmental anionic liposome/cationic polymer complexes in these cases, which are based on electrostatic interactions, similarly as salt screens electrostatics in polyelectrolyte complexes [34]. Eventually, this can lead to their decomplexation and their subsequent disassembly.

**Fig 1:**
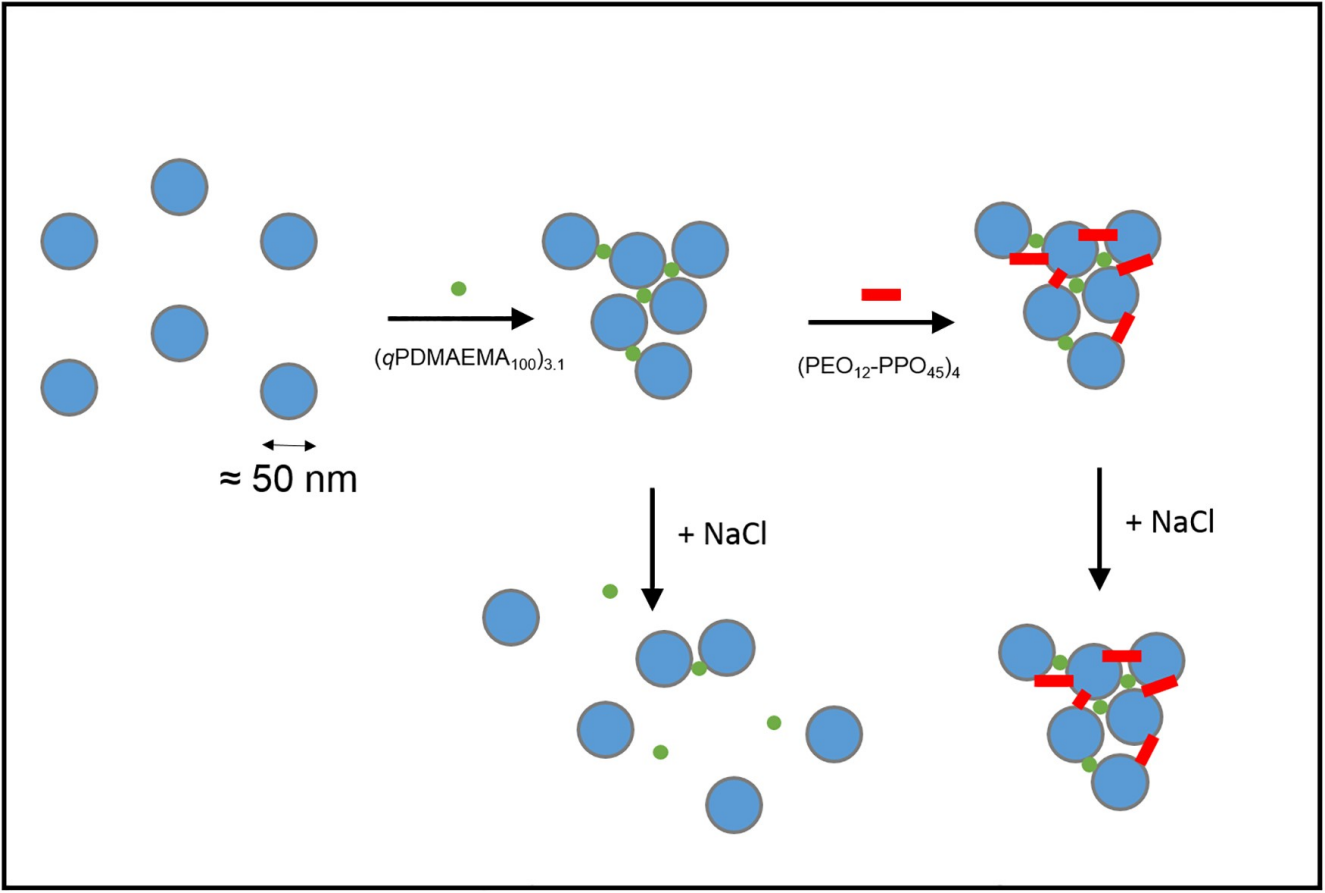
Schematic view on liposome “Gluing”. Synergistic effect of amphiphile and polyelectrolyte on their complexation with anionic liposomes and on the promoted structural stability of the resulting complexes upon salt addition.

As illustrated in Fig 1, we endeavor stabilizing these liposomal aggregates against salt screening with the addition of a multivalent (four-armed) star-shaped polymer being composed of four amphiphilic diblock arms containing an outer oil-soluble poly(propylene oxide) and an inner water-soluble poly(ethylene oxide) block: (PEO_12_-*b*-PPO_45_)_4_ (the indices assign the number average degree of polymerization and average arm number). PPO is water soluble and oil-soluble at low temperature, while at higher temperatures the water-solubility is lost due to its lower critical solution temperature LCST behavior [35]. This leads to a facile preparation of the polymer solutions without time-consuming solvent exchanges or rehydration techniques by just dissolving the polymer in cold water. The size of the resulting complexes was investigated by dynamic light scattering when adding amphiphilic polymer solutions to liposomes or to preformed liposome/polycation aggregates [36]. Likewise, the salt-dependent size of the complexes was investigated. The multiliposomal character of the complexes was tested by two-focus fluorescence correlation spectroscopy. Keeping in mind the carrier role of liposomes, the liposomal integrity was further assessed after the addition of PEO-PPO star shaped polymers by conductometry.

## Materials and methods

**Fig 2:**
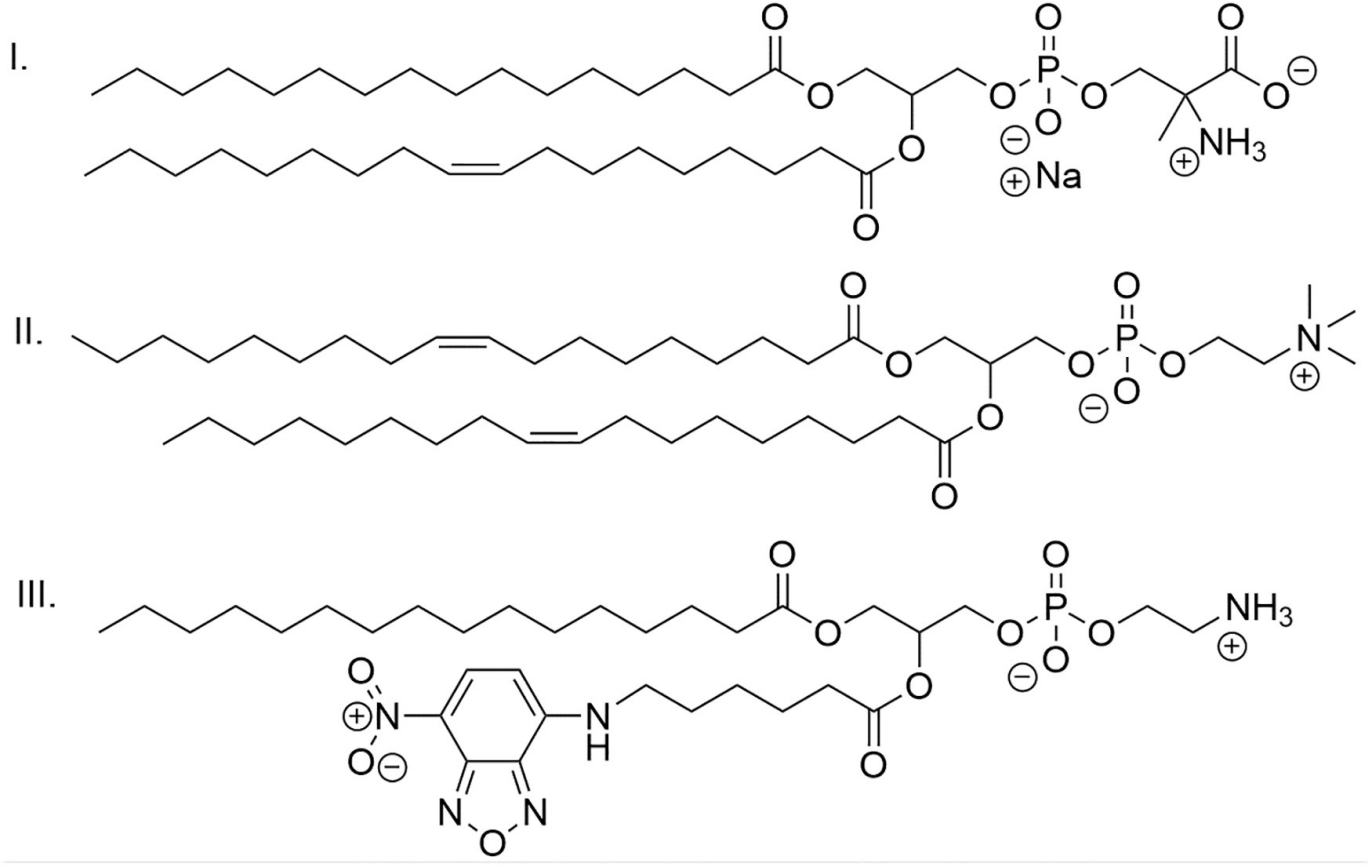
Chemical structures of lipids used. I. POPS, II. DOPC, III. NBD PE.

Small unilamellar anionic liposomes (50 nm in diameter) were prepared by evaporation and sonification method [37, 38]. DOPC (zwitterionic 1,2-dioleoyl-*sn*-glycero-3-phosphocholine), purchased by Avanti lipids USA, was dissolved in chloroform:ethanol (1:1 v:v) and POPS (anionic 1-palmitoyl-2-oleoyl-*sn*-glycero-3-phospho-L-serine), purchased by Avanti Lipids USA was dissolved in chloroform (Fig 2). The molar fraction of anionic lipids can be described as *ν* = [POPS]/([POPS]+[DOPC]) = 0.1. Subsequently, the organic solvent was removed on a rotary evaporator (typically by drying 2 mL of a 10 mg/mL organic lipid solution). The resulting lipid film was dispersed usually in 2 mL Tris buffer (pH 7, 10^−2^ mol/L) by sonication during 2 times 5 minutes (with a break between both) with the ultrasonic homogenizer Bandelin Sonopuls HD 60 (at 80% power during continuous sonication), yielding a stock solution of liposomes (10 mg/mL), which was further diluted (0.01 mL of a stock solution filled up to total sample volume of 2 mL with the respective buffer/salt/polymer solutions) to reach a final lipid concentration of 0.05 mg/mL (except where otherwise stated; 0.05 mg/mL corresponds to 6.37 10^−6^ mol/L of negative charges). For the conductivity measurements, the liposome preparation was altered: the lipid film was resolvated and dispersed in Tris buffer (pH 7, 10^−2^ mol/L) containing sodium chloride (4 mol/L). Subsequent dialysis against pure buffer led to a dispersion of NaCl-loaded liposomes. For two-focus fluorescence correlation spectroscopy (2fFCS) measurements, a fluorescence-labelled lipid is added during the preparation of the liposomes, the 1-palmitoyl-2-(6-[(7-nitro-2-1,3-benzoxadiazol-4-yl)amino]hexanoyl)-*sn*-glycero-3-phosphoethanolamine (NBD PE from Avanti; Fig 2). 20 μL of NBD PE lipids (1mg/mL) is added to the DOPC and POPS. Approximately 0.1% of the lipids are labelled with the dye being incorporated into the membrane of the liposomes. The liposomes were stored always only for a short time (max. 2 weeks) to avoid the fusion of their membranes, which could reduce the reproducibility of the experiments.

**Fig 3:**
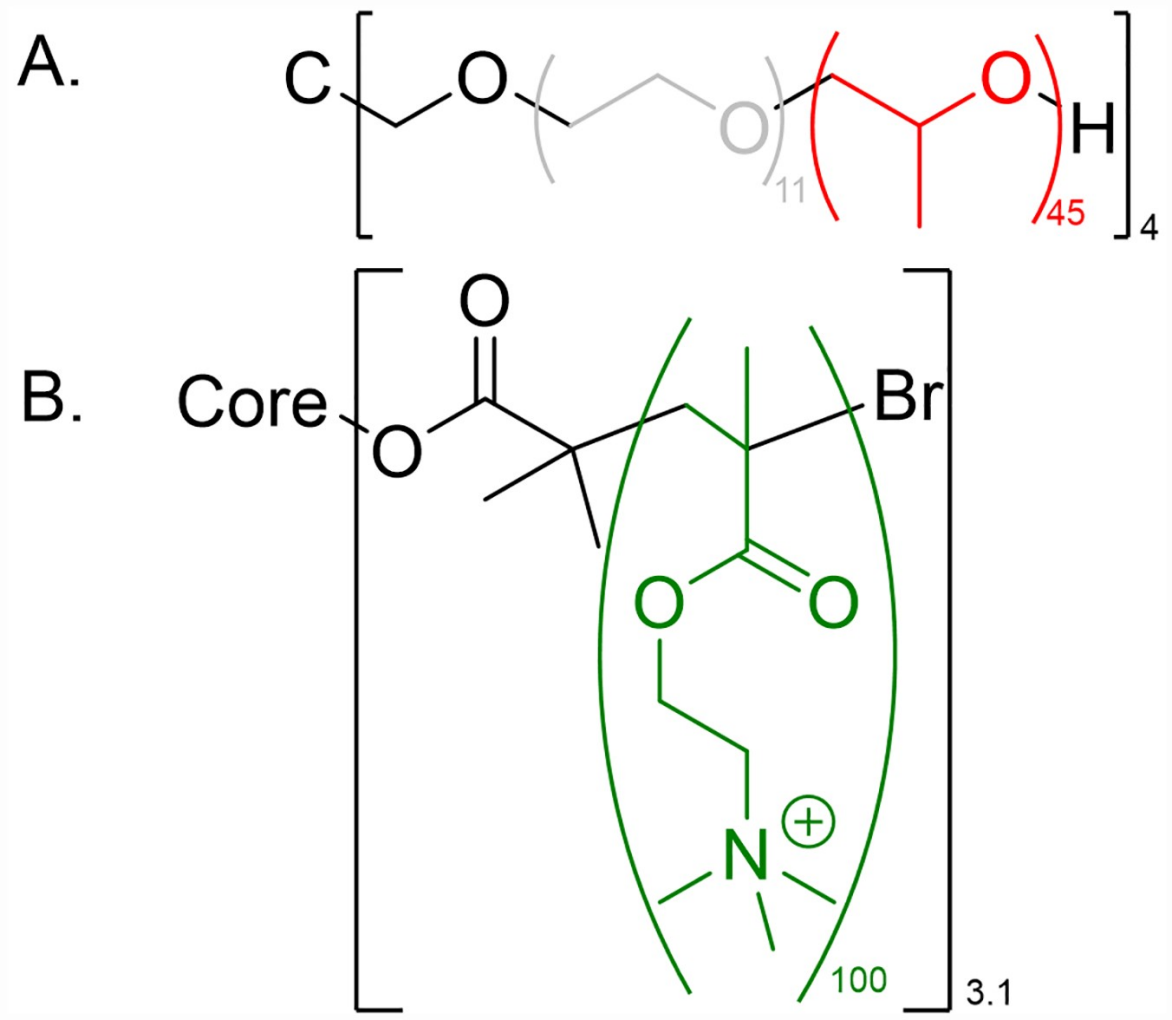
Chemical structures of polymers used. A. (PPO_45_-b-PEO_12_)_4_, B. (qPDMAEMA_100_)_3.1_ with glucose core (red: PPO polymer segments interacting with interior liposome membrane; grey: hydrophilic “non-interacting” PEO segments; green: polymer segments interacting with the exterior liposome membrane (charged lipids).

The cationic star-shaped polymer composed of 3.1 arms of quaternized poly[2-(dimethylamino)ethyl methacrylate] (*q*PDMAEMA_100_)_3.1_, also named poly([2-(methacryloyloxy)ethyl] trimethylammonium iodide) (PMETAI_100_)_3.1_ was already used in a previous paper (the indices assign the number average degree of polymerization and average arm number; Fig 3) [39]. We choose this polycation due its ability to build rather small multicompartmental liposome/polymer complexes with a limited number of polycation binders [33]. The star-shaped polymer composed of poly(propylene oxide) and poly(ethylene oxide) (PPO_45_-*b*-PEO_12_)_4_ was purchased from Polymer Source, USA (Fig 3). Sodium chloride (NaCl) was purchased by Sigma Aldrich, Germany, the ultra-pure Tris was bought from MP Biomedicals LLC, USA and hydrochloric acid was delivered from VWR Chemicals, Germany.

In all cases, double-distilled water was used, and the resulting buffer solution was filtered (RC, 0.20 μm) to remove large particles before being used for the dispersion of liposomes and polymers. The quantity of cationic polymers is set by the charge ratio Z = [+]/[–] = 0.3 of the molar concentration of positive charges of the polymers against the molar concentration of negative liposome charges. This ratio was motivated by previous results, where smaller multiliposomal aggregates were obtained [33]. By shaking, all the components were mixed. For the study of the size as a function of concentration of the amphiphilic star-shaped polymer, the preparation of the samples were carried out as follows: the polymers and the liposomes were mixed at 0 °C and stirred, for one day in an ice bath and an additional two days at room temperature since the critical micellization temperature *cmt* of (PEO_12_-*b*-PPO_45_)_4_ is around 20°C (S1 File, page 2 and 3). The first step (the stirring at 0 °C) does not seem to be essential for the formation of the complex (the polymer is well dissolved below 16 °C according to S1 File, page 2), therefore this step was skipped thereafter. The liposomes and the polymers were stored in the refrigerator at 5°C. The preparation of the different mixtures was carried out at room temperature by stirring. To enhance reproducibility, the stirring time was adjusted to one or two days at room temperature to allow for the incorporation of PPO into the membrane.

The hydrodynamic radii of liposomes and complexes were determined by dynamic light scattering (DLS) at 20 °C on an ALV setup equipped with a 633 nm HeNe laser (JDS Uniphase, 35 mV). Measurements were recorded in pseudo-cross correlation. The measurements were performed at nine different angles and the hydrodynamic radius was derived by help of the Stokes-Einstein equation from the diffusion coefficient obtained by a linear regression of the decay rate (from a second order cumulant fit) vs. the squared length of the scattering vector *q*^2^ [40].

Fluorescence correlation spectroscopy (FCS) is a non-invasive technique to investigate dynamic processes in solutions of low-concentration down to single particles or molecules [41–44]. The experiments were carried out on a MicroTime200 FCS setup from PicoQuant. Using an inverse confocal microscope (IX71, Olympus), laser light is focused into the sample volume exciting the fluorophores. The emission of fluorescent molecules within the focus is collected by two single-photon avalanche detectors (SPAD). Measuring the emitted light over time and the subsequent correlation of the resulting signals gives access to information on the corresponding dynamic processes, in particular on diffusion. For the two-focus FCS (2fFCS) method applied here, two laser beams of the same wavelength (470 nm), but with perpendicular polarization are coupled into the microscope. Using a polarization sensitive Nomarski prism, the two beam paths are slightly shifted apart resulting in two laterally shifted but overlapping foci within the sample volume [45, 46]. The diffusion coefficient, and subsequently the hydrodynamic radius, can be determined by fitting the measured data with appropriate theoretical models. It is linked to the fluorescently labelled entity. In case of labelled liposomes, the hydrodynamic radius gives information on the aggregation state of the liposomes only. The fitting procedure of experimental 2fFCS data is carried out according to the theoretical background as introduced by Dertinger et al. [45] For the evaluation, including correlation and fitting, an implementation as MATLAB script was kindly provided by the research group of Prof. Jörg Enderlein, Georg-August-Universität Göttingen, Germany. The distance between the two foci, the shift distance δ, is an intrinsic parameter of the system and depends among other things on the laser wavelength. It is not a fitting parameter but was determined beforehand from reference measurements of samples with known diffusion coefficient (here: ATTO 488 in water) and serves as intrinsic ruler.

We employed cryogenic transmission electron microscopy (cryo-TEM) to visualize the liposome complexes. Rapid vitrification in liquid ethane of the samples was used to investigate the complexes in their native (dispersed) state. Therefore, a vitrobot system was employed at 22 °C at 100% humidity. 4 μL of a dispersion were transferred onto a hydrophilized Lacey carbon coated TEM grid and vitrified. The grids were transferred into a Carl Zeiss Libra 120 microscope operating at a voltage of 120 kV with a bottom mounted CCD camera. We observed the zero-loss energy-filtered transmission electron microscopy images. Liposome aggregates were observed with cryo-TEM, which are in the same size range as observed by DLS.

## Results and discussion

In this part, different aspects are examined: 1. The size evolution of multiliposomal complexes, which are formed by addition of (PPO_45_-PEO_12_)_4_ and incorporation of PPO residues into different liposome membranes. 2. The retention or the loss of the integrity of the complexes. 3. The effects of salt on size/aggregative stability of the complexes.

**Fig 4:**
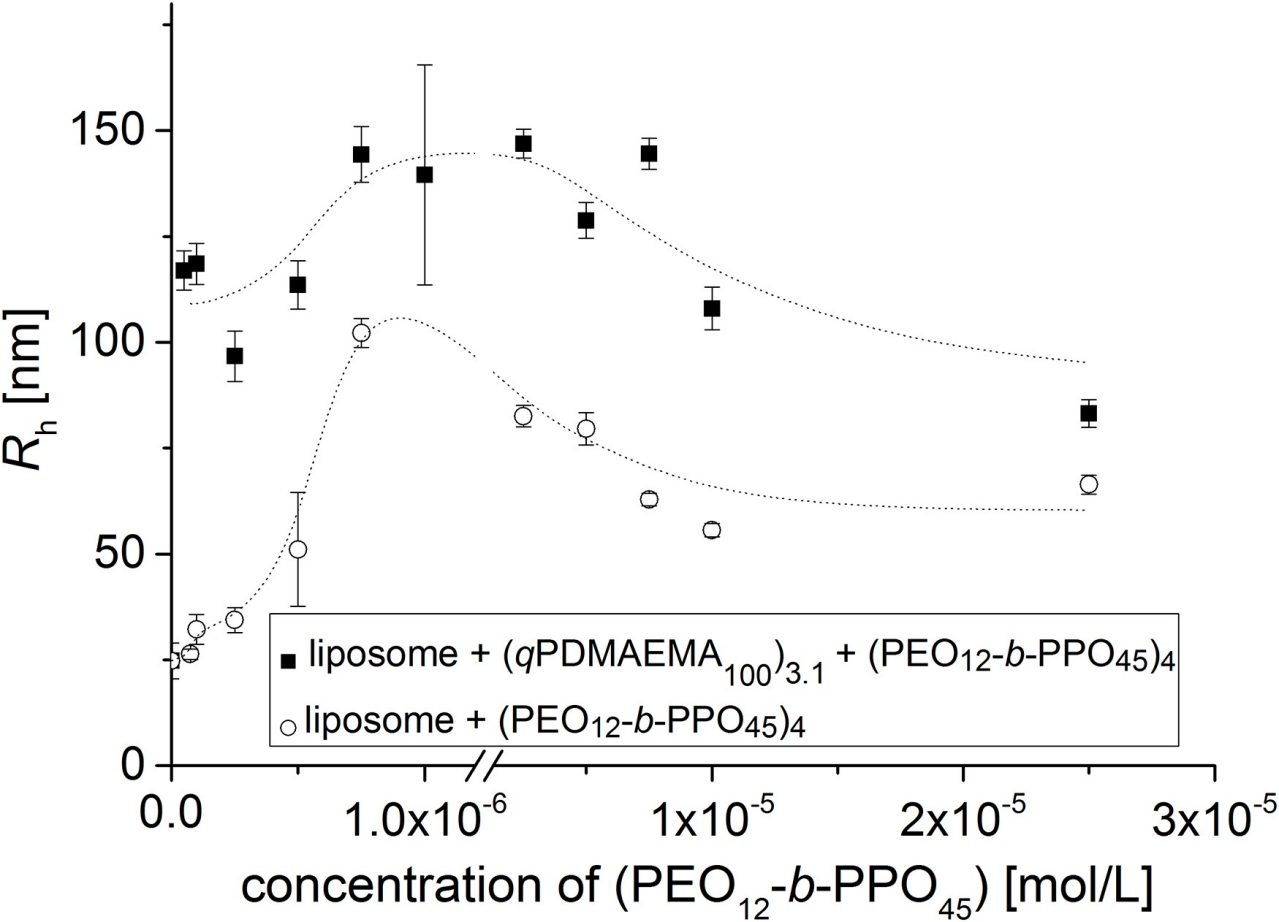
Course of size with varying “Glue” concentration. The hydrodynamic radii for the liposome/(PEO_12_-b-PPO_45_)_4_ polymer complexes and the liposome/(qPDMAEMA_100_)_3.1_/(PEO_12_-b-PPO_45_)_4_ polymer complexes at different (PEO_12_-b-PPO_45_) concentrations. The lipids concentration is 6.37 x 10^−5^ mol/L. For one data set, the concentration of the positive charges of (qPDMAEMA_100_)_3.1_ was held constant at 1.9 x 10^−6^ mol/L. Tris Buffer pH = 7, 20°C (lines are a guide to the eye).

Fig 4 shows the evolution of the size of the system with the addition of the (PEO_12_-b-PPO_45_)_4_ star-shaped polymer. The graph is plotted against the concentration of arms of the star-shaped polymer (PEO_12_-b-PPO_45_)_4_, because this concentration can be easily compared to the concentration of exterior lipids, sitting at the outside of the liposomes (for more information, see the S1 File, page 3).

**Fig 5:**
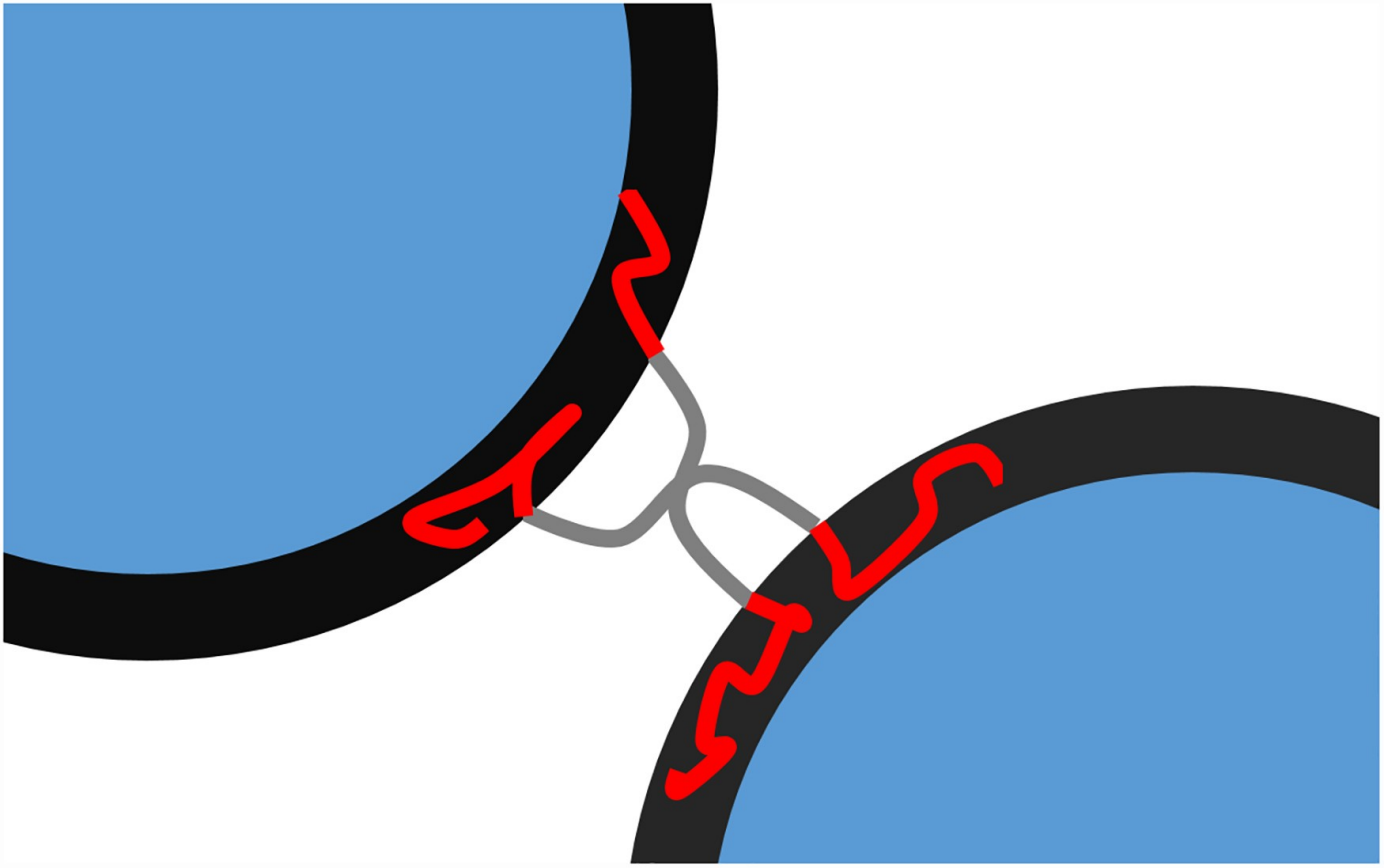
Gluing mechanism. Simplified scheme of the interaction between the PEO-PPO star-shaped polymer and two liposomes. The PPO block is represented in red and the PEO block in grey.

Two systems are studied: liposomes mixed with (PEO_12_-*b*-PPO_45_)_4_ and liposomes mixed with (*q*PDMAEMA_100_)_3.1_ and (PEO_12_-*b*-PPO_45_)_4_. For low concentrations of (PEO_12_-*b*-PPO_45_)_4_, the size of both systems increases with addition of (PEO_12_-*b*-PPO_45_)_4_ star-shaped polymer. There are two possible explanations for this behaviour: (1) it could be in line with a (homo)aggregation of the polymer in addition to single liposomes or liposome/(*q*PDMAEMA_100_)_3.1_ complexes non-interacting with the amphiphilic copolymer, leading to an increased average hydrodynamic radius. Alternatively, (2) the increase in size could indicate a facilitated aggregation of liposomes, which is in line with the interaction of PPO and the hydrophobic membrane of the liposomes (complexation): the hydrophobic part of the star (PPO) penetrates the lipid membrane to stay in the hydrophobic area whereas the hydrophilic part of the star (PEO) stays in the buffer (Fig 5).

**Fig 6:**
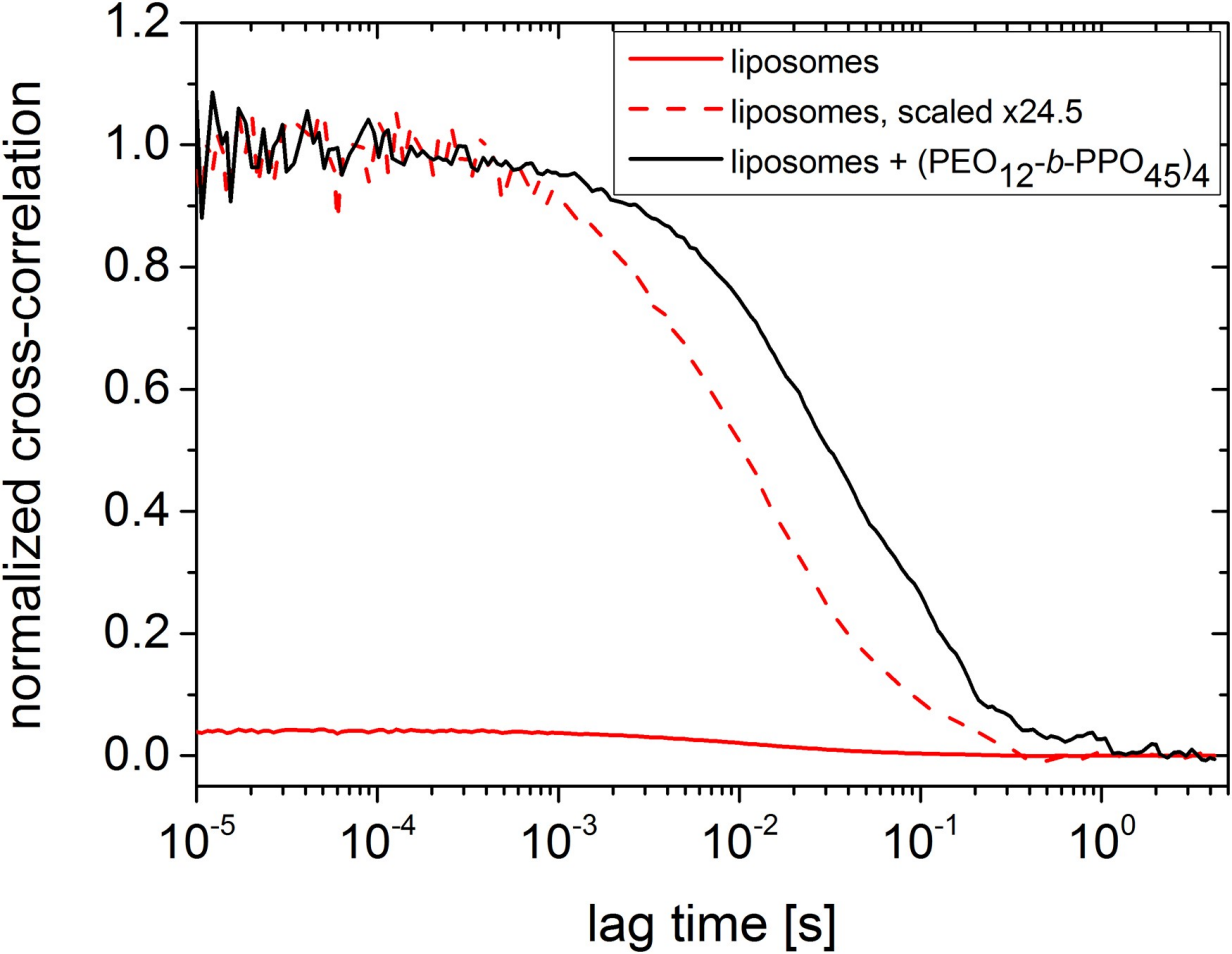
Demonstration of gluing. 2fFCS cross-correlation curves of lipids labelled with nitrobenzoxadiazole (NBD).[47] Liposomes (red curves) and complexes liposome/(PEO_12_-*b*-PPO_45_)_4_ (black curve) without NaCl. For a direct comparison, also the curve of the liposome scaled to the curve of the complex liposome/(PEO_12_-*b*-PPO_45_)_4_ is presented as red dotted line. The lipid concentration for each sample was 6.37 x 10^−5^ mol/L. The concentration of (PEO_12_-*b*-PPO_45_)_4_ was 7.5 × 10^−7^ mol/L. 10^−2^ mol/L Tris buffer was used at pH = 7, 20 °C.

To investigate whether the size change discussed above is caused by (homo)aggregation of the polymer or by the facilitated aggregation of liposomes, we utilized two-focus fluorescence correlation spectroscopy (2fFCS). 2fFCS is sensitive to the fluorescently labelled entities and its aggregation state. The increase of the size by introducing (PEO_12_-*b*-PPO_45_)_4_ was demonstrated by using lipids, which are labelled with nitrobenzoxadiazole (NBD) as fluorescence dye [47]. The liposomes were prepared with the same procedure but NBD-labelled lipids are added to the standard lipids. During the preparation, we assume a homogeneous distribution of the dye in the membrane of the liposomes. The FCS cross-correlation curves of the liposomes are presented in Fig 6 (red curves). Addition of (PEO_12_-*b*-PPO_45_)_4_ causes an increase in lag time indicating an increase of the size of the aggregates (Fig 6, black curve). Hence, the liposomes aggregate to multicompartmental containers when the amphiphilic polymer is added, though the size distribution of aggregates, which are formed by the complexation of (PEO_12_-*b*-PPO_45_)_4_ with the liposomes, seems large. The hydrodynamic radius of the (PEO_12_-*b*-PPO_45_)_4_/liposome complex is determined via the Stokes-Einstein equation. The size of the complex is approximately *R*_h_ ≈ 160 nm whereas the size of the liposomes is approximately 30 nm both being comparable with or only slightly larger than the DLS results. The differences can be explained by slightly different history of the preparation of the 2fFCS samples (see experimental section). For complete 2fFCS correlation curves and more information about the time trace of the 2fFCS, see S1 File, page 5/6.

**Fig 7:**
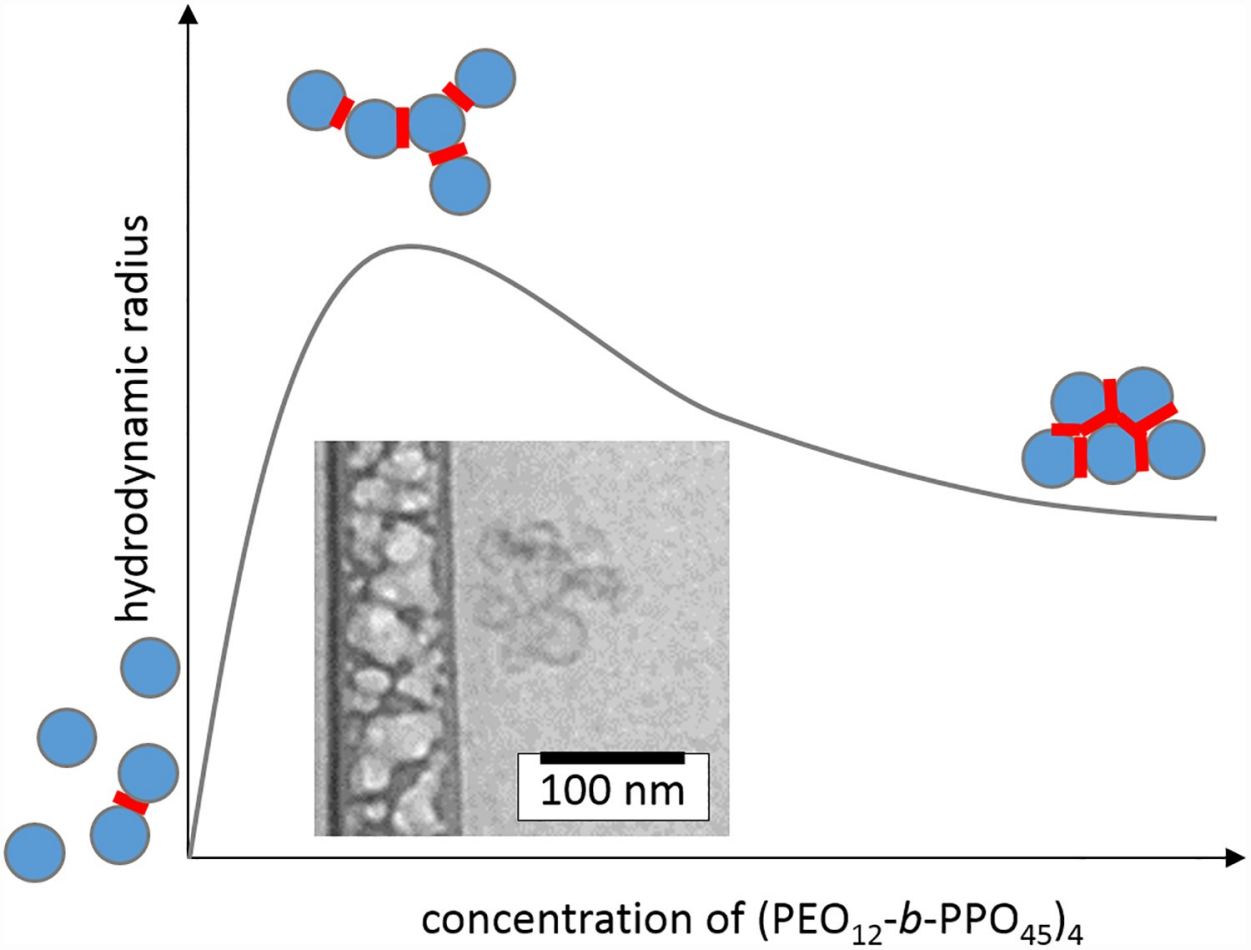
Schematic evolution during gluing with illustration of liposome/glue aggregate. Size of the complex liposomes/(PEO_12_-b-PPO_45_)_4_ with the increase of concentration of (PEO_12_-b-PPO_45_)_4_ star shaped polymer, including a cryo-TEM image of liposome/(PEO_12_-b-PPO_45_)_4_ complexes (scale bar 100 nm; the liposome concentration is 0.05 mg/mL; the concentration of (PEO_12_-b-PPO_45_)_4_ is 7.5 × 10^−7^ mol/L; temperature 20 °C, 10^−2^ mol/L Tris Buffer, pH = 7).

Hence, we conclude that the size increase in Fig 4 is caused by the aggregation of liposomes, induced by the gluing effect of (PEO_12_-*b*-PPO_45_)_4_, until the concentration of arms reaches 7.5 x 10^−7^ mol/L. The multiliposomal character is also corroborated by cryo-TEM (see Fig 7). After this concentration, the size decreases but stays bigger than the size of individual liposomes. Complexes/aggregates are still present, but the average size is smaller. With increasing amount of (PEO_12_-*b*-PPO_45_)_4_, the complexes become probably more compact and the hydrodynamic radius decreases (Fig 7).

In presence of (*q*PDMAEMA_100_)_3.1_, the electrostatically induced preaggregation leads always to larger multicompartmental aggregates also in presence of the amphiphilic star, which further “glues” a limited number of preaggregates probably by a similar mechanism as discussed for the mere (PEO_12_-*b*-PPO_45_)_4_ / liposome interaction (Fig 7). In addition, the surface of the (*q*PDMAEMA_100_)_3.1_/liposome aggregates might get saturated with (PEO_12_-*b*-PPO_45_)_4_, whose hydrophobic arms all immerse eventually into the same liposome, leading to a return toward the original size.

The choice of the concentration of the star-shaped polymer is very important because it determines the size of the complexes and its surface. The number of liposomes per complex determines the carrier capability. This number can be roughly estimated with the ratio of volume of the complex, considered as a sphere, to the volume of the liposomes multiplied by the packing factor (0.74 for closely packed spheres):

$$number\;of\;liposomes=\frac{volume\;of\;complex}{volume\;of\;liposome}*0.74$$ (1)

At elevated concentration of PEO-PPO star shaped polymer (10^−5^ mol/L), the complex liposome/PEO-PPO contains approximately 10 liposomes on average and the complex liposome/polycation/PEO-PPO contains approximately 70 liposomes on average according to the formula (1), which gives only a theoretical approximation. To reach this result, we ignore any dispersity in size, the actual packing and the size of the polymer because of the small actual volume occupied by the polymer outside the liposomes.

**Fig 8:**
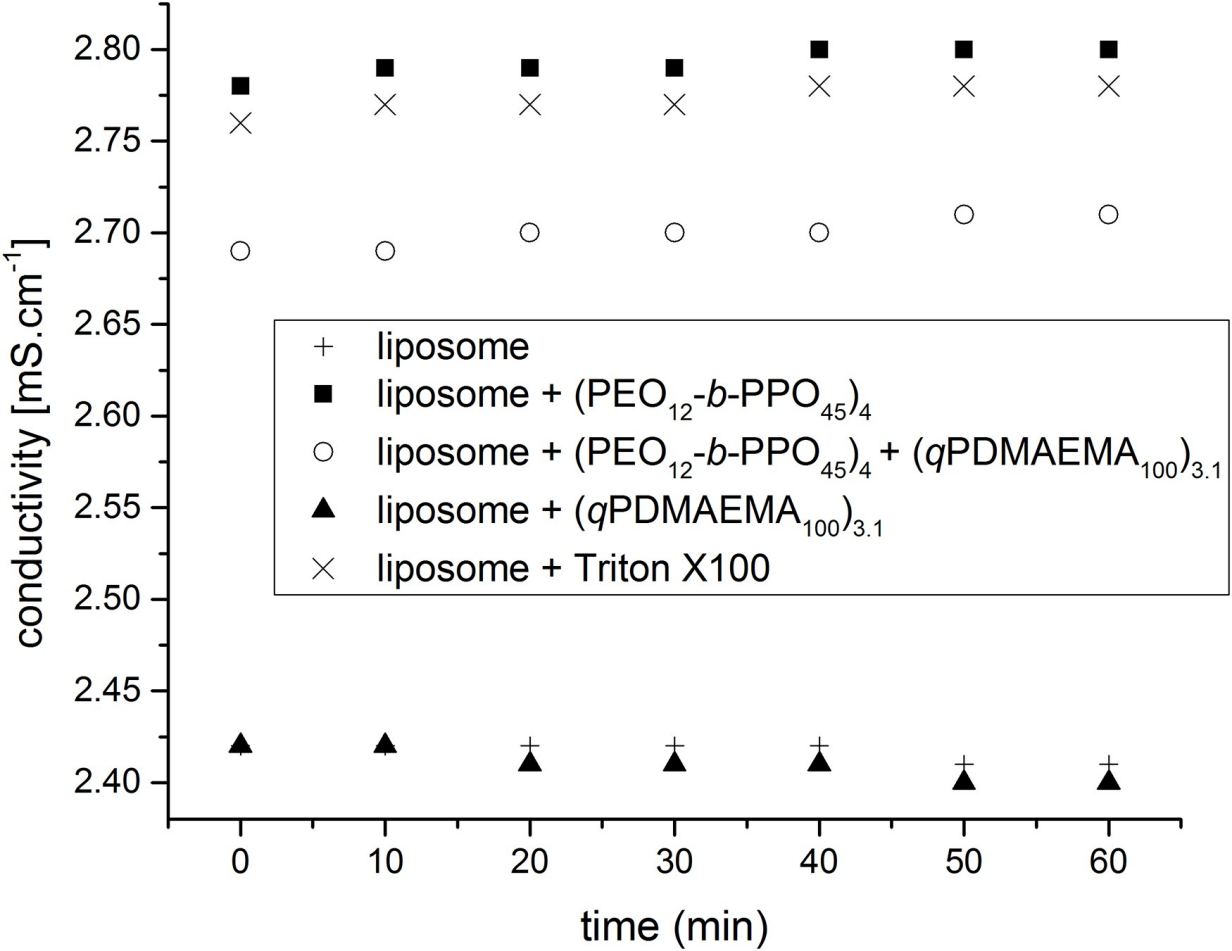
Integrity evaluation. Conductivity of solutions with liposome/polymer complexes vs. time, the liposomes are loaded with 4 mol/L NaCl and 10^−2^ mol/L Tris buffer. The lipid concentration is 1.1 10^−3^ mol/L for each sample. The concentration of PEO_12_-*b*-PPO_45_ arms for the respective samples is 1.8 10^−4^ mol/L. If present, the concentration of the positive charges of (*q*PDMAEMA_100_)_3.1_ is 3.31 10^−4^ mol/L, it corresponds to Z = 0.3. Tris Buffer pH = 7, 20°C.

At least a considerable number of liposomes within the complexes should keep their membrane integrity to entrap the cargo within the multiliposomal containers. To test the cargo entrapment, the liposomes are loaded with a sodium chloride solution at 4 mol/L and the time-dependent conductivity is measured (Fig 8). Contrary to the DLS measurements, conductivity measurements are better conducted in more concentrated solution (compare to Fig 4). The complex is at maximum size at 7.5 x 10^−7^ mol/L PEO_12_-b-PPO_45_ for the concentration of 6.4 10^−5^ mol/L for the liposomes. We can see that the complex of liposomes and (PEO_12_-b-PPO_45_)_4_ does not retain its membrane integrity. The conductivity is indeed close to the conductivity of the liposomes/Triton X100 solutions, i.e. the conductivity is the same as the one for opened liposomes. The conductivity of the liposomes/(qPDMAEMA_100_)_3.1_/(PEO_12_-b-PPO_45_)_4_ complexes is higher than the conductivity of the pure liposomes but still lower than the conductivity of the opened liposomes. The reason is not totally clear yet, but apparently some synergistic effects come into play for the mixtures of polycationic and amphiphilic stars. It can be probably explained by the fact that some liposomes in the middle of the complex are not easily penetrated by the poly(propylene oxide), which do not lose their integrity. We estimate with Eq (1) that for the complexes of 70 liposomes, approximately 20 liposomes retain their integrity (a conductivity increase by 0.38 mS/cm corresponds to 0% integrity, while the observed 0.28 mS/cm increase results in 26% integrity). They can still be used as part of multi-containers for hydrophilic substances. In any case, the ability of these capacious multiliposomal containers for delivery of hydrophobic substances is expected to be unaffected by the presence of (PEO_12_-b-PPO_45_)_4_, as the amphiphilic star does not considerably influence the overall liposomal shape within the multiliposomal construct (Fig 7). Though the overall hydrophilic loading capability is reduced (which could be further tailored by membrane modification with e.g. cholesterol), the synergistic action of both stars allows the further use of these complexes as multicomponent delivery vehicle, especially when the resulting complexes would exhibit an increased salt tolerance.

**Fig 9:**
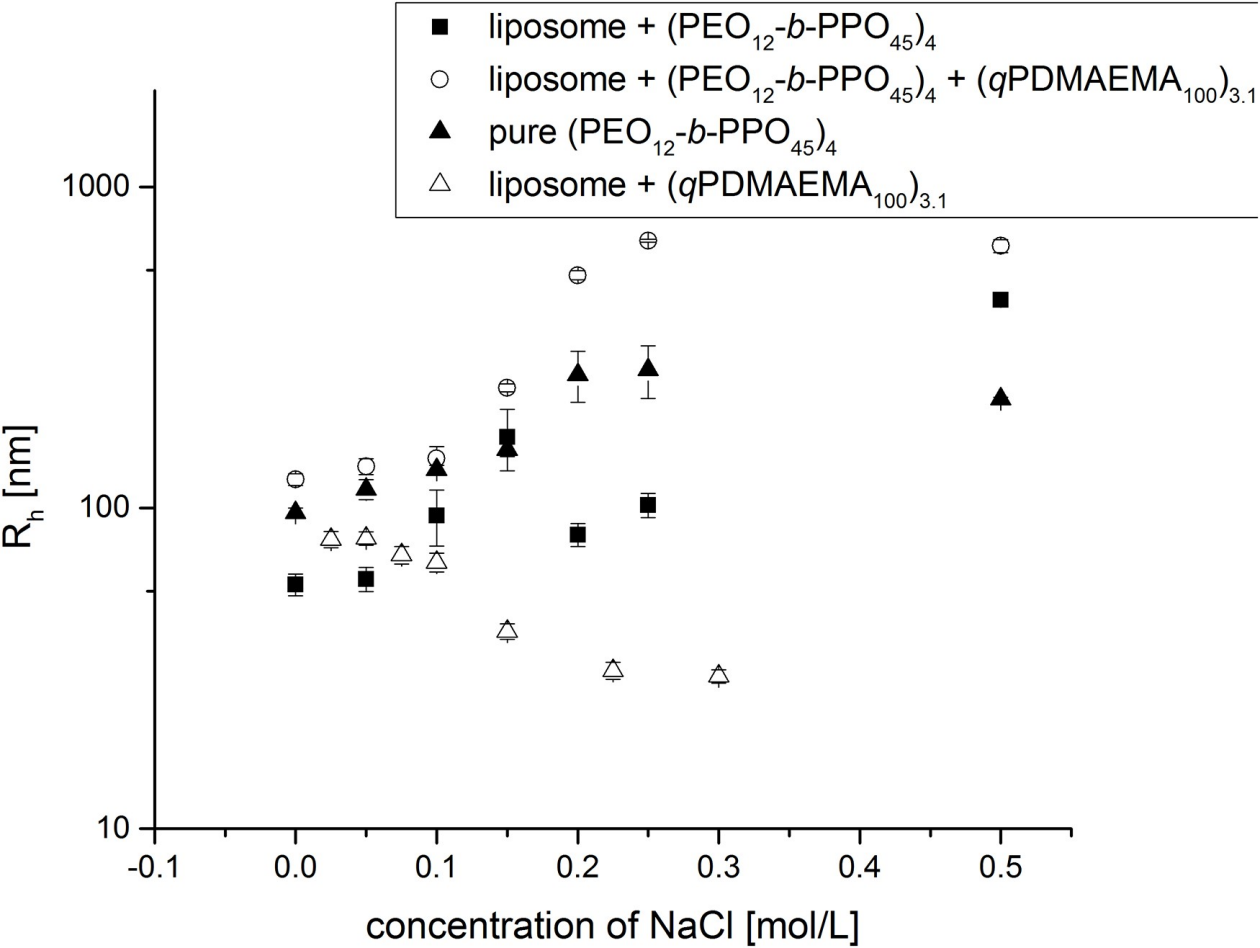
Salt stability enhancement. Hydrodynamic radii of the (PEO_12_-b-PPO_45_)_4_, the liposome/(PEO_12_-b-PPO_45_)_4_ complexes, the liposome/(qPDMAEMA_100_)_3.1_/(PEO_12_-b-PPO_45_)_4_ complexes and the liposome/(qPDMAEMA_100_)_3.1_complexes at different NaCl concentrations. The lipid concentration for each sample is 6.37 x 10^−5^ mol/L. Where relevant, the concentration of the positive charges of (qPDMAEMA_100_)_3.1_ was held constant at 1.9 10^−6^ mol/L (which correspond to a charge ratio Z = 0.3, the liposomes are in excess). The concentration of (PEO_12_-b-PPO_45_) is 7.5 10^−7^ mol/L. Tris Buffer pH = 7, 20°C.

Indeed, the mere liposome/(*q*PDMAEMA_100_)_3.1_ complex disassembles in presence of sodium chloride (Fig 9 and S1 File, page 7). This is a problem for e.g. prospective drug delivery, where it is important that the complex stays stable at physiological conditions, i.e. at 0.15 mol/L of sodium chloride. Fig 9 shows the evolution of the size with increase of the salt concentration. As reference, the size of the pure (PEO_12_*-b*-PPO_45_)_4_ is determined as well, showing increasing size with increasing concentration of sodium chloride. Here, we are interested only the relative size changes, without a detailed addressing of the self-assembly behavior of the amphiphilic star. Indeed, the amphiphilic polymer loses solubility when the salt concentration increases, which is in line with a further salting-out effect of the PPO groups. This behaviour is also inherited to the liposome complexes, as the trend is the same for the liposome/(PEO_12_*-b*-PPO_45_)_4_ complex and the liposomes/(qPDMAEMA_100_)_3.1_/(PEO_12_*-b*-PPO_45_)_4_ complex. For a salt concentration of 0.15 mol/L, the complex is already bigger than without salt. This can be also explained by the reduced electrostatic repulsion between the anionic liposomes, which makes the bridging by the amphiphilic star polymer even more effective. In addition, the increase in size can be taken as an advantage because more containers will be attached in the same complex. Hence, an increased stability of the multiliposomal constructs against electrostatic screening (by salt) and disintegration toward single liposomes can be assured by using the amphiphilic copolymer.

## Conclusion

We prepared aggregates of liposomes linked together by an amphiphilic star-shaped polymer and in some cases additionally by an oppositely charged polyelectrolyte. The hydrophobic stickers at the end of the arms of the amphiphilic polymer interact with the inner membrane (i.e. lipid tails) of several liposomes, while the oppositely charged polyelectrolyte leads to an additional electrostatic “gluing” of lipid head groups of various liposomes. It was demonstrated that the size of the liposome aggregates increases due to a fluffy interconnection of the liposomes at rather low concentrations of the amphiphilic polymer “glue” [48]. However, the structure of the liposome aggregates is compacted at higher concentrations of the amphiphilic polymer. Though the presence of amphiphilic polymer is detrimental to the membrane integrity, the liposome aggregates do not disassemble at high salt concentrations (which is the case for mere electrostatic binding). Even more, the combined action of polyelectrolyte and amphiphilic polymer leads synergistically to salt-resistant aggregates with a still considerable integrity of the membrane. By using the described approach, a multi-liposomal container with especially high capacity to hydrophobic substances (like drugs [49]) could be established.

## Supporting information

S1 File Supporting_Information.pdf. Critical micelle temperature investigations, a discussion on the ratio of PPO vs lipids, count traces obtained during Fluorescence Correlation Spectroscopy including further autocorrelation functions, an investigation on the additional salt influence and further references.
(PDF)

S1 Data Collection.zip. Data sets presented in the manuscript.
(ZIP)
