# Supplementary material for: PEO-b-PPO star-shaped polymers enhance the structural stability of electrostatically coupled liposome/polyelectrolyte complexes
Source: PLoS One. 2019 Jan 17;14(1):e0210898. doi: 10.1371/journal.pone.0210898 (PMC6336312; doi:10.1371/journal.pone.0210898)
Supplement: S1 File — Critical micelle temperature investigations, a discussion on the ratio of PPO vs lipids, count traces obtained during Fluorescence Correlation Spectroscopy including further autocorrelation functions, an investigation on the additional salt influence and further references. (PDF) [file pone.0210898.s001.pdf]

Supporting information

PEO-*b*-PPO star-shaped polymers enhance the structural stability of electrostatically coupled liposome/polyelectrolyte complexes

Camille E. Pinguet,[1] Esther Ryll,[1] Alexander A. Steinschulte,[1] Jón M. Hoffmann,[1] Monia Brugnoni,[1] Andrey Sybachin,[2] Dominik Wöll,[1] Alexander Yaroslavov,[2] Walter Richtering,[1] Felix A. Plamper,[1,3]*

[1]Institute of Physical Chemistry, RWTH Aachen University, Aachen, Germany

[2]Department of Chemistry, M.V. Lomonosov Moscow State University, Moscow, Russian Federation

[3]Institute of Physical Chemistry, TU Bergakademie Freiberg, Freiberg, Germany

1. Critical micelle temperature 2
2. Ratio PEO-PPO arm/lipids 3
3. Enhancement of hydrophobic domain size 4
4. Fluorescence Correlation Spectroscopy 5
5. Influence of sodium chloride 7
6. Supporting References 8

1. Critical micelle temperature

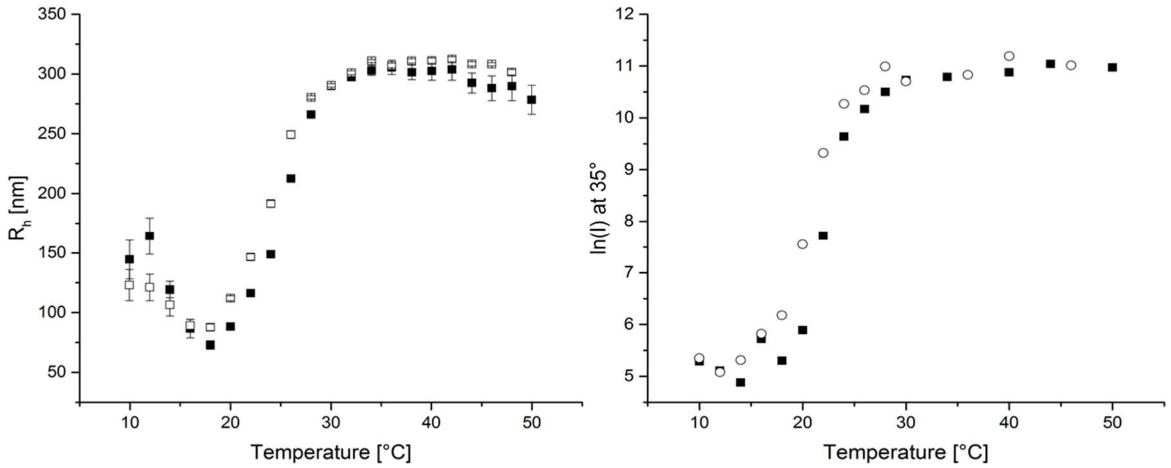

Figure S1. The hydrodynamic radii and scattering intensity at 35° of the star-shaped polymer $(PEO_{12}$-*b*-$PPO_{45})_4$ at different temperatures. The concentration of $(PEO_{12}$-*b*-$PPO_{45})_4$ is 1.25×10^{-5} mol/L. The heating is represented by full squares, the cooling by empty squares.

The hydrodynamic radius of the star-shaped polymer $(PEO_{12}$-*b*-$PPO_{45})_4$ depends on the temperature. The hydrodynamic radius decreases from 10 to 18 °C (probably dominated by loose aggregates). Beyond 18 °C, it increases until circa 35 °C. The dependence of temperature on the size is due to a change of solubility with temperature. According to the evolution of the scattering intensity with temperature, the critical micelle temperature is estimated to be approximately 22 °C. This is in accordance to previous results, where the actual micellization occurred also around 20 °C for a linear PEO-*b*-PPO block copolymer.[1]

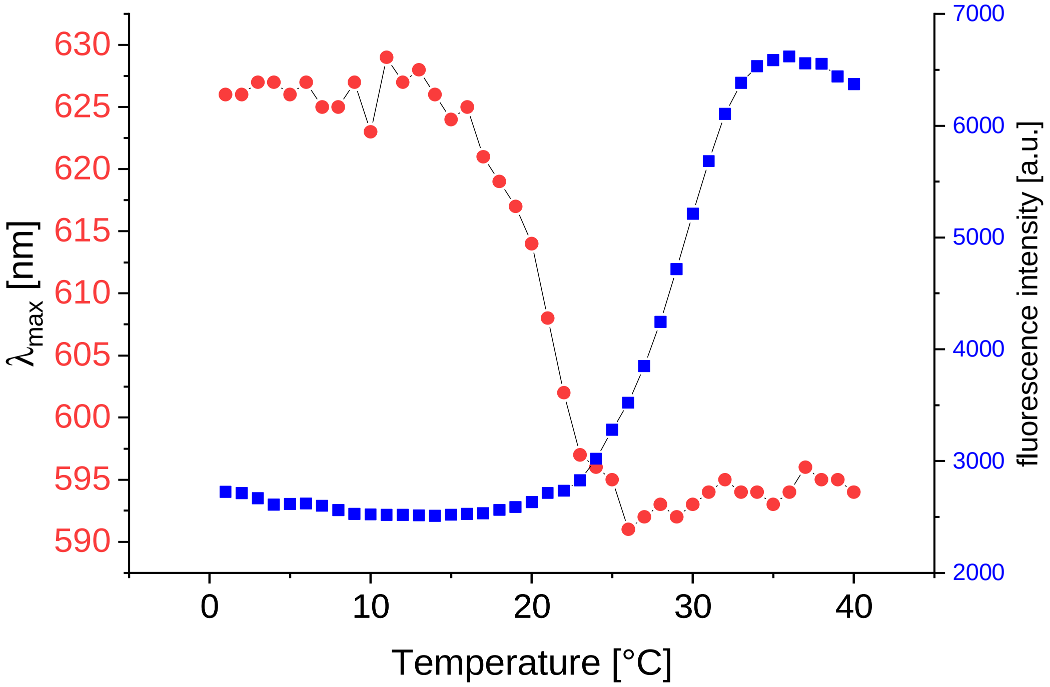

Figure S2. Intensity of the fluorescence (blue squares; integrated from 500 to 800 nm) and wavelength of the maximum intensity (red circles) as a function of the temperature (excitation wavelength λ_{ex} = 470 nm). The concentrations of $(PEO_{12}$-*b*-$PPO_{45})_4$ and Tris Buffer are 1.25×10^{-5} mol/L and 10^{-2} mol/L, respectively. The pH value is 7.

For such a linear PEO_{113}-*b*-PPO_{69}, formation of hydrophobic domains (unimolecular micelles) was observed already above approximately 5 °C using suitable fluorescent probes.[1] Below 5 °C, the polymer is hydrophilic due to the thermoresponsivity of PPO, while aggregation and micellization occurs above approximately 20 °C. Hence, we applied the same procedure for the star-shaped PPO/PEO polymer used in this study. The probing dye 4-(dicyanomethylene)-2-methyl-6-(4-dimethylaminostyryl)-4H-pyran (4-HP) was purchased from Sigma-Aldrich and used as received. 4-HP was dissolved in THF at 5 mg/mL. 0.5 µL of this solution is added to 20 mL Tris Buffer (10^{-2} mol/L, pH = 7); in this solution of 4-HP (at 0.5 × 10^{-6} mol/L), $(PEO_{12}$-b-$PPO_{45})_4$ was added and the fluorescence was measured during excitation at 470 nm. The intensity of fluorescence was plotted against temperature. The intensity of the peak increases with the microviscosity surrounding the dye[2] and hence with the polymer aggregation state (the hydrophobic dye partitions into the hydrophobic domains, which get more viscous upon formation of dense aggregates).[1] The aggregated character of $(PEO_{12}$-b-$PPO_{45})_4$ becomes apparent from 22 °C, which is in strong agreement with the conclusion observed in Figure S1. Likewise, the peak location (at the maximum of fluorescence intensity), which is a measure of the hydrophobicity of the dye surrounding, is plotted against temperature. The change of wavelength for the maximum of the intensity corresponds already to a change from a hydrophilic to a hydrophobic dye environment (from 625 nm in pure brine to below 600 nm in polymer solution with hydrophobic domains) at around 16 °C, being in line with previous observations.[1] Hence, unimolecular micelles are formed above 16 °C, which aggregate above 20 °C. The differences to the previously reported results on PEO_{113}-*b*-PPO_{69} can be explained by the shorter PPO block in the star and the different polymer topology (star polymer vs. block copolymer).

2. Ratio PEO-PPO arm/lipids

The number of lipids at the exterior and at the interior can be calculated as follows:

$$N_{ext} = \frac{4\pi\left(\frac{d}{2}\right)^2}{a} \quad (1)$$

$$N_{int} = \frac{4\pi\left(\frac{d}{2}-h\right)^2}{a} \quad (2)$$

Where d is the diameter of the liposome, h the thickness of the lipid bilayer (about 5 nm) and a the surface of the head of the lipid (about 0.71 nm^2). This quantity of lipid at the exterior of the liposome is for a radius of 25 nm estimated to be at 60% of the total lipids concentration.

In Figure 2 (main part), the hydrodynamic radius of the complexes increases until the concentration of arms reaches 7.5 × 10^{-7} mol/L, when there is one arm for approximately 52 surface lipids.

3. Enhancement of hydrophobic domain size

The hydrophobic domain can be favorably enlarged to enhance the hydrophobic cargo capacity by use of multiliposomal adducts compared to a mere size increase of a single liposome. This is seen by comparing the total surfaces for both cases, while keeping the overall dimensions R_{max} constant. We consider a larger single liposome with the radius R_{max}, volume $V_{max} = \frac{4\pi}{3} \cdot R_{max}{}^3$ and surface $A_{max} = 4\pi \cdot R_{max}{}^2$. This larger liposome can be divided into x portions (i.e. smaller liposomes) by assuming a certain packing fraction $P \approx 0.74$. Then one of the smaller liposomes possesses a volume $V_{min} = \frac{P}{x} \cdot \frac{4\pi}{3} \cdot R_{max}{}^3 = \frac{4\pi}{3} \cdot R_{min}{}^3$. This yields as radius of the smaller liposomes $R_{min} = \sqrt[3]{\frac{P}{x}} \cdot R_{max}$. All these liposomes exhibit now a total surface $A = x \cdot 4\pi \cdot R_{min}{}^2 = x \cdot 4\pi \cdot \left(\frac{P}{x}\right)^{2/3} \cdot R_{max}{}^2$. This means that the surface (and thereby the hydrophobic domain size) is enhanced during the division into smaller liposomes by the factor

$$\frac{A}{A_{max}} = x^{1/3} \cdot P^{2/3} \qquad (3)$$

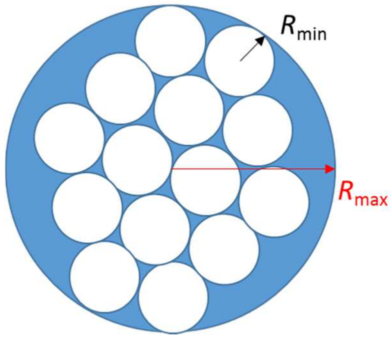

Figure S3. Geometry for calculating the increase in hydrophobic domain size by subdivision of a larger liposome into smaller liposomes

4. Fluorescence Correlation Spectroscopy

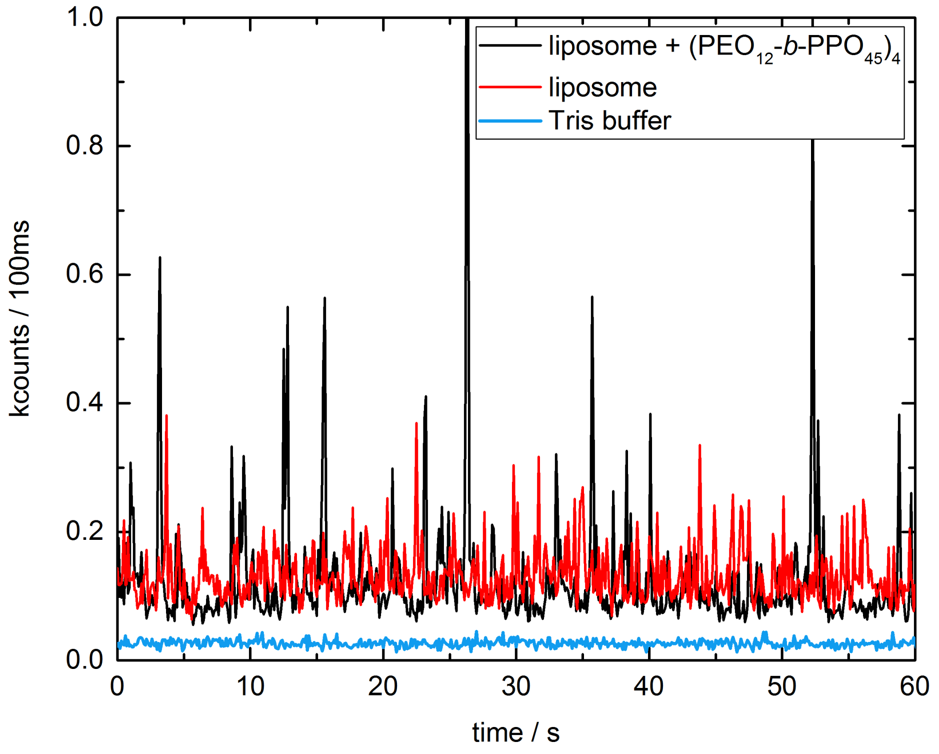

Figure S4. Representative time traces of lipids labelled with nitrobenzoxadiazole (NBD). Liposomes (red curve), complexes liposome/$(PEO_{12}$-*b*-$PPO_{45})_4$ (black curve) and Tris buffer (blue curve) are measured without NaCl. The lipids concentration for each sample is 6.37×10^{-5} mol/L. The concentration of $(PEO_{12}$-b-$PPO_{45})_4$ is 7.5×10^{-7} mol/L. 10^{-2} mol/L Tris buffer was used at pH = 7. Temperature 20 °C.

Figure S4 shows the intensity time traces of FCS measurements of three different sample systems. The number of measured photons (in thousand counts per 100 milliseconds) of the first 60 seconds of these measurements are plotted. The intensity trace of the buffer (blue), which does not include any fluorophore, gives the background level and shows no distinct peaks as expected. The intensity trace of the liposomes (red) shows an overall increased count rate with frequent occurrences of smaller intensity bursts with a maximum of around 400 counts per 100 ms. This shows the presence of labeled liposomes. The intensity trace of the sample with complexed liposomes shows these frequent, smaller bursts as well. However, more striking are the less frequent but more intense bursts with a maximum of around 2.67 kcounts/100ms. These originate from the formed complexes, which are remarkable brighter due to accumulation of more fluorophores and allow for a clear distinction between systems with individual liposomes and larger complexes.

Figures S5 and S6 show the auto- and cross-correlation curves of samples with pure liposomes and liposomes with added $(PEO_{12}$-*b*-$PPO_{45})_4$, respectively. The diffusion coefficient of pure liposomes, as determined in the first measurement is used as an additional fixed value during the fit of the second system with added $(PEO_{12}$-*b*-$PPO_{45})_4$.

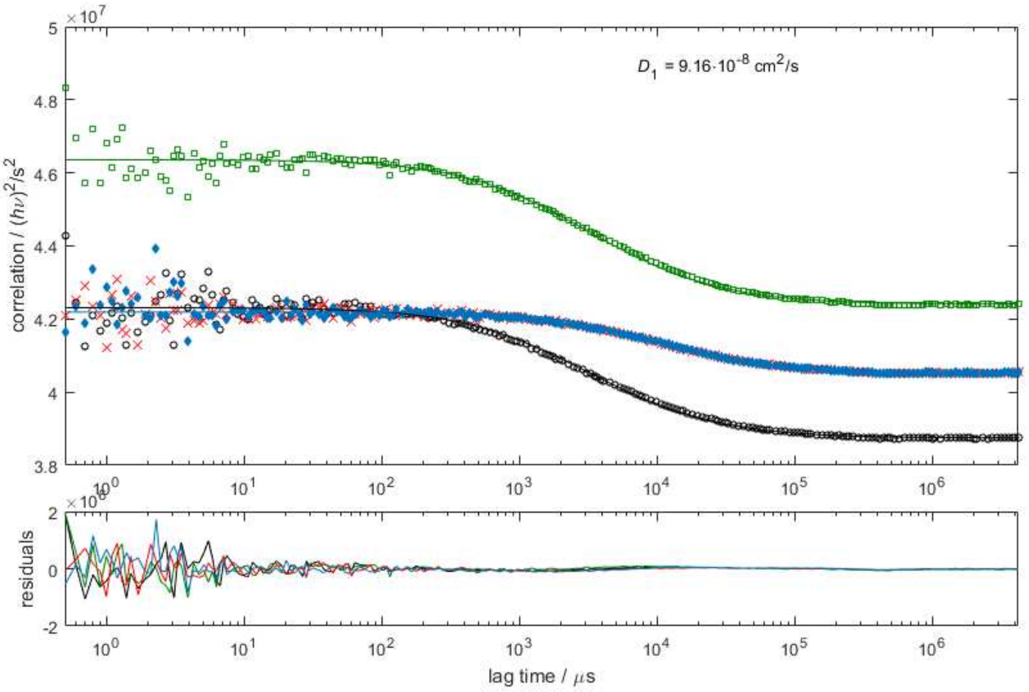

Figure S5. 2fFCS of pure liposomes. Auto-correlation data of first (black open circles) and second (green open squares) focus, cross-correlation curves between focus 1 and 2 (red crosses) and reverse (blue diamonds). Related fits are depicted as lines. The calculated diffusion coefficient of liposomes equals $D_1 = 9.16 \times 10^{-8}$ cm^2 s^{-1}.

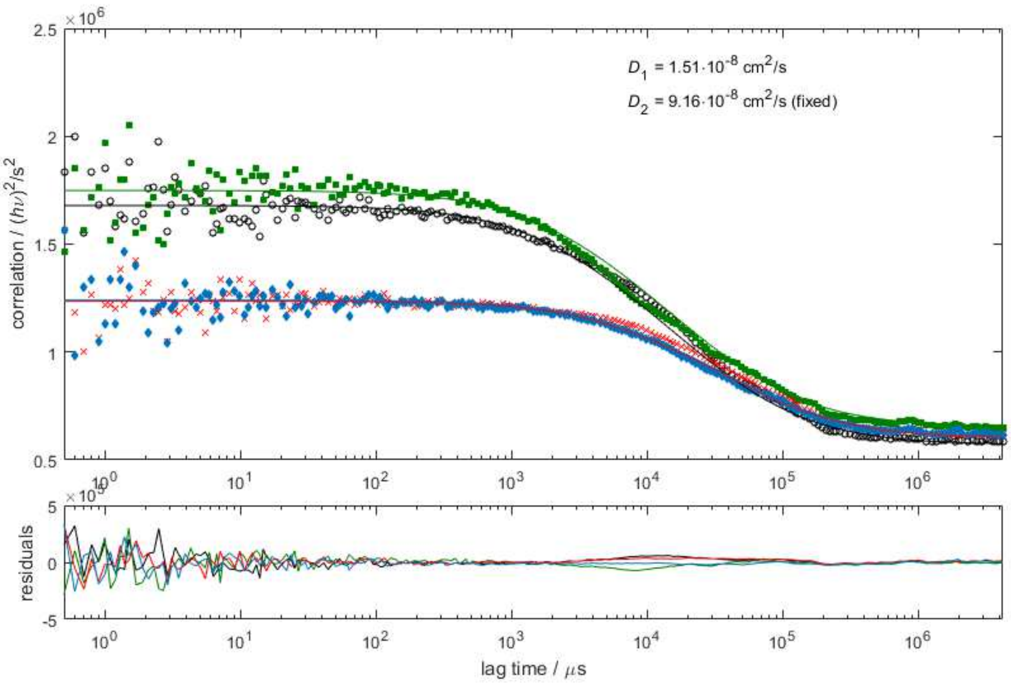

Figure S6. 2fFCS of liposomes with added $(PEO_{12}$-*b*-$PPO_{45})_4$. Auto-correlation data of first (black open circles) and second (green open squares) focus, cross-correlation curves between focus 1 and 2 (red crosses) and reverse (blue diamonds). Related fits are depicted as lines. Calculated diffusion coefficient of complexes equals $D_1 = 1.51 \times 10^{-8}$ cm^2/s with fixed diffusion coefficient component of free liposomes of $D_2 = 9.16 \times 10^{-8}$ cm^2/s.

5. Influence of sodium chloride

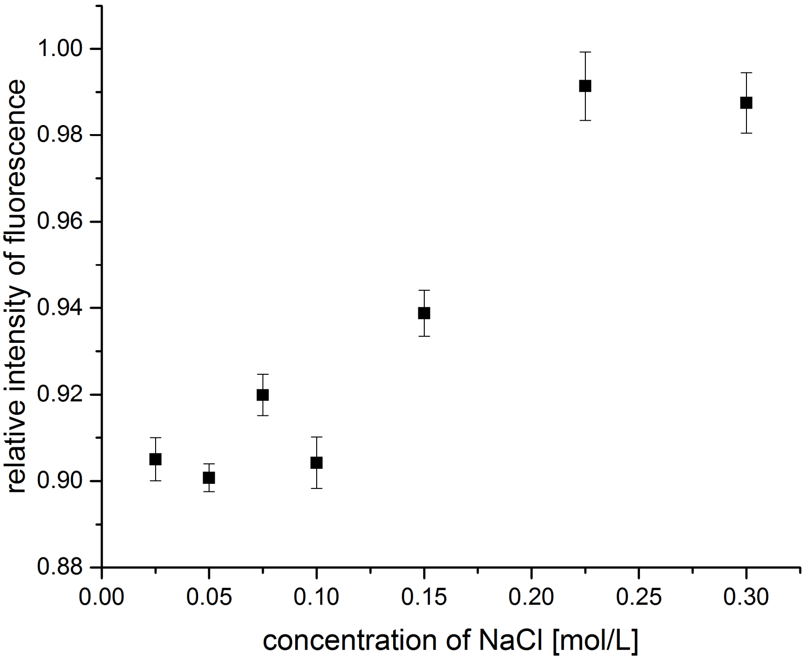

Figure S7. Relative intensity of fluorescence of the liposome/(qPMAEMA$_{100}$)$_{3.1}$ complexes at different sodium chloride concentrations. The lipids concentration for each sample is 6.37 × 10^{-5} mol/L. The concentration of positive charges of (qPMAEMA$_{100}$)$_{3.1}$ is 1.9 × 10^{-5} mol/L. It correspond to a charge ratio Z = 0.3.

To confirm that the complexes are disassembled at high NaCl concentrations, the fluorescence intensity was investigated as a measure for light scattering. The liposomes were prepared as usual (see in the experimental section) but the pyrene-labelled lipid 1,2-dioleoyl-*sn*-glycero-3-phosphoethanolamine-*N*-(1-pyrenesulfonyl) lipid (as received from Avanti Lipids, USA; Figure S8) was added to POPS and DOPC. The fraction of pyrene-labelled lipid was 0.2 wt%. At 20 °C, the fluorescence intensity for excitation at λ_{ex} = 352 nm was measured at λ_{em} = 380 nm. The relative intensity was calculated by dividing the fluorescence intensity of the complex (with (qPMAEMA$_{100}$)$_{3.1}$) by the fluorescence intensity of the free liposomes (without (qPMAEMA$_{100}$)$_{3.1}$). The complexation-induced aggregation increases the turbidity of the medium, and therefore also causes a loss in the relative intensity of fluorescence observed by the detector.[3] For low salt concentrations, the relative fluorescence intensity is lower due to scattering of the aggregates as shown in Figure S8. For increasing salt concentrations, the aggregates disassemble and the reduced scattering recover the fluorescence intensity to its level of the free liposomes.

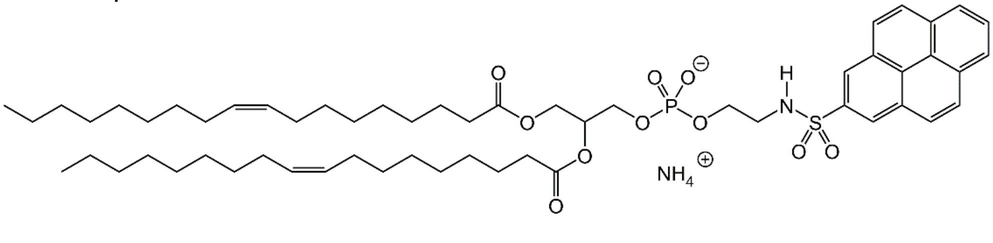

Figure S8. Chemical structure of pyrene-labelled lipid.

6. Supporting References

[1] Steinschulte, A. A.; Schulte, B.; Rütten, S.; Eckert, T.; Okuda, J.; Möller, M.; Schneider, S.; Borisov, O. V.; Plamper, F. A., Effects of Architecture on the Stability of Thermosensitive Unimolecular Micelles. *Phys. Chem. Chem. Phys.* **2014,** *16* (10), 4917–4932.

[2] Virtanen, J.; Lemmetyinen, H.; Tenhu, H., Fluorescence and EPR studies on the collapse of poly(N-isopropyl acrylamide)-g-poly(ethylene oxide) in water. *Polymer* **2001,** *42* (23), 9487–9493.

[3] Pinguet, C. E.; Hoffmann, J. M.; Steinschulte, A. A.; Sybachin, A.; Rahimi, K.; Wöll, D.; Yaroslavov, A.; Richtering, W.; Plamper, F. A., Adjusting the size of multicompartmental containers made of anionic liposomes and polycations by introducing branching and PEO moieties. *Polymer* **2017,** *121*, 320–327.
